# Asclepain cI, a proteolytic enzyme from *Asclepias curassavica* L., a south American plant, against *Helicobacter pylori*

**DOI:** 10.3389/fmicb.2022.961958

**Published:** 2022-08-18

**Authors:** Ángel Gabriel Salinas Ibáñez, Anabella L. Origone, Constanza S. Liggieri, Sonia E. Barberis, Alba E. Vega

**Affiliations:** ^1^Laboratorio de Microbiología e Inmunología, Facultad de Química, Bioquímica y Farmacia, Universidad Nacional de San Luis, San Luis, Argentina; ^2^Instituto de Física Aplicada (INFAP) - Centro Científico Tecnológico (CCT) San Luis - Consejo Nacional de Investigaciones Científicas y Técnicas (CONICET), San Luis, Argentina; ^3^Laboratorio de Control de Calidad y Desarrollo de Bromatología, Universidad Nacional de San Luis, San Luis, Argentina; ^4^Centro de Investigación de Proteínas Vegetales (CIProVe), Universidad Nacional de La Plata, La Plata, Argentina

**Keywords:** asclepain cI, *Asclepia curassavica* L. (Asclepiadaceae), safe nutraceutical product, antimicrobial proteolytic enzyme, *helicobacter pylori*, natural therapeutic adjuvant

## Abstract

*Helicobacter pylori* is a Gram negative bacterium most frequently associated with human gastrointestinal infections worldwide. The increasing occurrence of antibiotic-resistant isolates of *H. pylori* constitutes a challenge. The eradication of the microorganism is currently being considered a “high priority” by the World Health Organization (WHO). In this context, bioactive compounds found in natural products seem to be an effective therapeutic option to develop new antibiotics against the pathogen. In this study, we investigated the effect of asclepain cI, the main purified proteolytic enzyme of the latex of petioles and stems from *Asclepia curassavica* L. (Asclepiadaceae), a South American native plant, against *H. pylori*; in order to obtain a natural therapeutic adjuvant and a safe nutraceutical product. Asclepain cI showed antibacterial activity against reference strains and drug-resistant clinical isolates of *H. pylori in vitro*. A range of minimal inhibitory concentration (MIC) from 1 to 2 μg/ml and minimal bactericidal concentration (MBC) from 2 to 4 μg/ml was obtained, respectively. The action of asclepain cI on the transcription of *omp*18, *ure*A, *fla*A genes showed a significantly decreased expression of the selected pathogenic factors. Furthermore, asclepain cI did not induce toxic effects at the concentrations assayed. Asclepain cI could be considered a highly feasible option to be used as a natural therapeutic adjuvant and a safe nutraceutical product against *H. pylori*.

## Introduction

*Asclepias curassavica* L. (Asclepiadaceae), which is also locally known as flor de sangre (blood flower), bandera española (Spanish flag), algodoncillo (scarlet milkweed), platanillo or hierba Maria (Marie herb), is an erect evergreen perennial subshrub with a woody base, that grows up to 1.6 m tall (Asclepiadaceae, 2022). The plant is native to the South American biographical region although it has become a naturalized weed in tropical and subtropical areas around the world (Floridata, 2022). The dull green/reddish stem is smooth and round. The 8–12 cm long and 5–24 cm wide leaves are simple, opposite, and lanceolate, with short petioles. The umbel-shaped inflorescence has 6–15 flowers that head on short axillary and terminal peduncles. Flowers are bright red or orange around a yellow center (Bendre and Kumar, 2014).

The roots, leaves, and stems of *A. curassavica* contain several biologically active molecules, such as flavonoids, triterpenes, polyphenols, proteins, carbohydrates, fixed oils, saponin, and steroids (Bate and Smith, 1962; Oliver-Bever, 1986; Hembing, 2000). After drying and decoction, the entire plant is used in traditional medicine for its anti-inflammatory, antimicrobial, anticancer, antithrombotic, antioxidant, and hemostatic properties (Moulin-Traffort et al., 1990; Shivaprasad et al., 2009a; Baskar et al., 2012; Lee et al., 2012; Yuan et al., 2016; Qamar et al., 2019; Zheng et al., 2019; Nakano et al., 2020; Alonso-Castro et al., 2021).

Two cysteine-type phytoproteases, asclepain cI and asclepain cII, were isolated, biochemically characterized, and then purified from petioles and stem latex. Both proteases were inhibited using cysteine proteases inhibitors like E-64, maximal proteolytic activity was exhibited at pH 8.0–8.5, which remained stable within that pH range. The proteases showed significant thermal stability at temperatures between 40 and 60°C but were completely inactivated at 70°C. The isoelectric points of both proteases were greater than 9.3. The highest endosterolytic activity on the synthetic substrates N-α-carbobenzoxy-p-nitrophenyl esters of amino acids was expressed with the glutamine derivative. Although asclepain cII is the minor proteolytic component of the enzymatic extract, it showed higher specific activity than asclepain cI (Liggieri et al., 2004, 2009).

Cysteine proteases from the Asclepiadaceae plants latex have exhibited thrombin and plasmin like activities (Shivaprasad et al., 2009b).

The primary scientific novelty of this work lies in the fact that there are no existing reports within the literature on the antibacterial activity of asclepain cI and cII or on the *Helicobacter pylori* activity of asclepain cI and cII.

*Helicobacter pylori* is a Gram-negative bacterium, which has been defined as a group I carcinogen since 1994. It is currently clinically associated with the most frequent human infections worldwide, such as gastritis, peptic ulcer, gastric adenocarcinoma, and mucosa-associated lymphoid tissue lymphoma (Ansari and Yamaoka, 2017; Asgari et al., 2018; Akeel et al., 2019; Nagata et al., 2020; Holmes et al., 2021).

The bacterium colonizes an exclusive ecological niche, the human stomach, through several pathogenic factors, like outer membrane proteins, urease, motility, chemotaxis, and its helical shape (Ansari and Yamaoka, 2019).

These factors allow *H. pylori* to move within the viscous gastric mucosa, facilitate its attachment to epithelial cells and counteract the acidic environment of the human stomach. Also, the production of carbon dioxide (CO_2_) and ammonia (NH_3_) by urea hydrolysis, provides a nitrogen source for the bacterium (Cheok et al., 2021; Woo et al., 2021).

The management of *H. pylori* infection includes triple or quadruple antibiotic therapy with clarithromycin (CLA), metronidazole (MTZ), or amoxicillin (AML) plus a proton pump inhibitor (Ogasawara et al., 2020). However, treatment failure is a global concern due to the increase in resistant strains (Kim et al., 2015; Marcus et al., 2016; Dang and Graham, 2017). In this sense, the WHO encourages the urgent search for safe and efficient compounds for the priority treatment of *H. pylori* and other multiresistant bacteria (Roszczenko-Jasińska et al., 2020).

Natural plant extracts provide a feasible alternative to either attack *H. pylori* targets or modulate the host's immune system (Salinas Ibáñez et al., 2017; Korona-Glowniak et al., 2020).

Studies carried out with polyphenols from wine, apple peel, or olive oil have shown that they can cause the rupture of the outer membrane of some bacteria and the decrease in the release of urease and adhesion factors such as SabA or Vac (Parreira et al., 2016). On the other hand, Brown et al. (2016) demonstrated that adverse environmental conditions can increase the transcription of the gene for the survival and virulence of the bacteria (Brown et al., 2016).

The group of proteolytic enzymes shows different structures, an affinity for substrate, and a reaction mechanism (Vallés and Cantera, 2018; Barcia et al., 2020). Thus, subtilisin proves to be effective against *Pseudomonas* sp, *Bacillus* sp, and *Listeria monocytogenes* (Longhi et al., 2008; Molobela et al., 2010). Lysostaphin is capable of breaking the pentaglycine bond between the peptidoglycan chains and lysing cell walls (Boksha et al., 2016); while pronase did not show an additive effect in eradicating *H. pylori* infection when combined with AML and CLA (Bang et al., 2015).

Adaro et al. observed that the partially purified proteolytic extract (≥50 μg of protein/ml) of the fruits from *Solanum granuloso-leprosum* (Dunal) decreased (*p* ≤ 0.05) the growth of *Escherichia coli* ATCC 25922. Nevertheless, no effect was observed against *Staphylococcus aureus* ATCC 25923 (Adaro et al., 2019). There is no further information in the literature regarding the use of native plant proteases with antibacterial activity against *H. pylori* (Bruno et al., 2003, 2006; Liggieri et al., 2004; Pardo et al., 2010; Cimino et al., 2015; Bersi et al., 2019).

The aim of the present study was to investigate the action of asclepain cI, the main purified protease of the latex of petioles and stems from *A. curassavica* L. (Asclepiadaceae) against *H. pylori*, and thus obtain a natural therapeutic adjuvant and a safe nutraceutical product.

## Materials and methods

### Plant material

Petioles and stems of *A. curassavica* L. (Asclepiadaceae), which grow in the city of La Plata, Province of Buenos Aires, Argentina, were collected. The latex was obtained by making incisions on the surface of both parts of the plant. The crude proteolytic extract was obtained by receiving the latex in cold 0.1 M citric phosphate buffer (pH 6.5) with 5 mM EDTA and 2 mM cysteine. This suspension was centrifuged at 16,000 *g* for 30 min at 4°C to remove gums and cell debris. Then, the supernatant was ultra-centrifuged at 100,000 *g* for 1 h at 4°C and kept at −20°C until purified (Ansari and Yamaoka, 2017).

### Purification of asclepain cI

The crude proteolytic extract was purified by cation exchange chromatography (FPLC). Two active fractions were isolated. The major purified protease (asclepain cI) showed a molecular mass of 23.2 kDa by mass spectrometry and a pI higher than 9.3 (Ansari and Yamaoka, 2017).

### Electrophoresis (SDS-PAGE)

Samples containing proteases were analyzed by sodium dodecyl sulfate-polyacrylamide gel electrophoresis (SDS-PAGE) using 12.5 % (w/v) polyacrylamide gels. The current was kept constant at 40 mA during stacking and then increased to 60 mA and kept constant for 40 min. Gels were stained with Coomassie Brilliant Blue R-250. Finally, protein purity was verified using the silver staining procedure (Ansari and Yamaoka, 2017). The electrophoretic profiles were analyzed by densitography using the latest version of ImageJ 1.31, Wayne Rasband of the Research Services Branch, National Institute of Mental Health, Bethesda, MD, USA.

### Protein concentration and specific proteolytic activity

The Bradford method was used for measuring the protein concentration of asclepain cI (Bradford, 1976). Proteolytic assays were performed during purification steps using 340 μl of 1 % w/v azocasein solution as substrate in a reaction mixture containing 340 μl of asclepain cI and 340 μl of 100 mM TRIS hydrochloride buffer (TCB) with pH 7.5 containing 15 mM cysteine. This buffer was also used for preparing substrate and enzyme solutions. The reaction was carried out for 10 min at 37°C, then stopped by the addition of 340 μl of 10 % w/v TCA. The mixture was centrifuged for 20 min at 15,400 × *g*, and the supernatant enzymatic activity was spectrophotometrically measured and defined in terms of the Azocaseinolytic Unit (Uazo). One Uazo is the amount of enzyme that produces an increase of one absorbance unit measured at λ: 337 nm after 1 min, under the test conditions. Negative control was performed by replacing the enzyme with buffer.

### Physicochemical properties of asclepain cI

#### Stability upon storage

Asclepain cI was stored for 24 months at −20°C. Its residual proteolytic activity was measured every 24 h using 0.5 ml of 10 mM N-alpha-benzoyl-DL-arginine-p-nitroanilide hydrochloride (BANI) (Sigma-Aldrich, USA) as substrate in a reaction mixture containing 0.5 ml of enzyme in 100 mM TCB pH 8 and 0.5 ml of 20 mM L-cysteine in 100 mM TCB pH 8. The reaction was carried out for 5 min at 37°C under 200 rpm of agitation. The absorbance was measured within the linearity range at λ: 410. Proteolytic enzyme activity was expressed in terms of international unit (IU). One IU was defined as the amount of enzyme that cleaves 1 μmol of BANI per min under previously defined conditions. A control was carried out by replacing the enzyme with buffer.

#### Solubility

Enzyme solubility is defined as the amount of soluble nitrogen in an aqueous solution or dispersion that is not sedimented by moderate centrifugal forces. The protein concentration (%) was determined by the Kjeldahl method (Kjeldahl, 1883). The performance of asclepain cI in emulsions, foams, and gels can be predicted by protein solubility, and it depends on pH, ionic strength, and temperature (Morr, 1985). Solubility is expressed as protein solubility index (PSI) (American Oil Chemists' Society, 1998), as follows (Equation 1):


(1) $$\begin{array}{l} \text{PSI}=\;\frac{\text{Protein content in the supernatant (mg/ml) }\times \text{ Volume of supernatant (ml)}}{\text{Sample weight (mg) }\times \text{ Protein content in the sample (mg/100 mg of sample) }}\times \text{ 100} \end{array}$$

#### Emulsifying properties

The emulsifying property of an enzyme is related to the amount needed to coat an interfacial area. Emulsifying activity index (EAI, m^2^/g) and emulsion stability index (ESI, min) were determined by using the turbidimetric technique as described by Pearce and Kinsella (1978) and modified by Tang et al. (2006). These indexes were calculated by means of Equations (2) and (3):


(2) $$\begin{array}{l} \text{EAI}=\;\frac{\text{2 }\times \text{ 2,303 }\times \text{ Ao }\times \text{ DF}}{\text{c }\times \;\phi \times \;\left(1-\theta \right)} \end{array}$$

(3) $$\begin{array}{l} \text{ESI}=\;\frac{A_{0}}{\left(A_{0}-A_{20}\right)}\;\times \text{20} \end{array}$$

Where:

c: is the protein concentration (mg/ml).

ϕ: is the optical path (0.01 m).

θ: is the fraction of oil used to form the emulsion.

DF: is the dilution factor.

#### Viscosity

A rheometer (Model DV-III, AMETEK Brookfield, MA, USA) was used for measuring the viscosity of asclepain cI (10 mg of protein/mL), from 100 to 140 rpm at 24°C. Mean viscosity was calculated (Miroslaw and Surówka, 2004).

#### Hydration properties

The amount of water that a protein can retain influences the formulation, processing, and storing of goods. Held water (HW, %) and water retention capacity (WHC, g of water/g of dry residue) of asclepain cI were determined under centrifugation at 3000 × *g* for 30 min at 20°C (Piva et al., 1981). HW and WHC were calculated by means of Equations (4) and (5).


(4) $$\begin{array}{l} \text{HW}=\;\frac{\text{weight of H2O in pellet}}{\text{weight of H2O in pellet }+\text{ weight of H2O in supernatant}} \end{array}$$

(5) $$\begin{array}{l} \text{WHC}=\;\frac{\text{weight of H2O in pellet}}{\text{weight of dry pellet}}\;\times \text{100} \end{array}$$

### Bacterial strains

In this study, we used *H. pylori* NCTC 11638 as a reference strain, which was supplied by Dr. Teresa Alarcon Cavero (Hospital Universitario de la Princesa; Madrid, Spain), and also 12 clinical strains isolated from patients of San Luis (Argentina). The characterization of the strains is shown in Table 1.

**Table 1:** Bacterial strains.

***H. pylori* strains**	**Antimicrobial susceptibility (mm)**			
	**AML**	**CLA**	**MTZ**	**LEV**
HP109	S	S	R	S
HP137	S	R	S	S
HP145	S	S	R	S
HP148	S	R	S	S
HP152	S	R	R	S
HP155	S	S	S	S
HP166	S	S	S	S
HP179	S	R	S	S
HP294	S	R	R	S
HP659	S	S	S	S
HP661	S	S	S	R
HP662	S	S	R	S
NCTC 11638	S	S	S	S

*R, resistant strains; S, sensible strains. Amoxicillin (AML, 10 μg), clarithromycin (CLA, 15 μg), metronidazole (MTZ, 5 μg) (Oxoid^TM^, Argentina), and levofloxacin (LEV, 5 μg) (Britania Laboratories, Argentina). The sensitivity of the strains was evaluated by the MIC breakpoint values, with ≤ 25 mm for AML (10 μg/ml), ≤ 28 mm for CLA (15 μg/ml), ≤ 18 mm for MTZ (5 μg/ml), and ≤ 18 mm for LEV (5μg/ml)*.

The strains were confirmed by gram staining, and positive biochemical reaction for urease, catalase, and oxidase. They were stored at −80°C in trypticase soy broth (TSB, Britania) supplemented with 20% glycerol (Biopack, Buenos Aires, Argentina). The antimicrobial sensitivity of the strains was determined by the MIC breakpoint values, according to the Clinical and Laboratory Standards Institute [Clinical and Laboratory Standards Institute (CLSI), 2010].

### Animals

BALB/c mice of 18 to 20 g of body weight were provided by the UNSL Animal Facility. The handling and care were carried out according to the norms of the Institutional Committee for the Care and Use of Animals (CICUA-UNSL) and the Guide for the Care and Experimental Use of Animals (DHEW publication NIH 80-23).

### Antibacterial activity of asclepain cI

The antibacterial activity of asclepain cI against *H. pylori* strains was evaluated using the broth microdilution method as previously described by Bang et al. (2015), Salinas Ibáñez et al. (2017). Asclepain cI concentration ranging from 32 to 0.125 μg of protein/ml was used and 2,3,5-triphenyl tetrazolium chloride (TTC, Merck KGaA, Darmstadt, Germany) solution was added as a viability indicator. Both positive and negative controls were included in all assays. Minimal inhibitory concentrations (MICs) were determined after 72 h of incubation at 37°C, as the lowest concentration of asclepain cI that inhibited microbial growth. Minimum bactericidal concentration (MBC) was defined as the least concentration of asclepain cI that prevented the growth of microorganisms on antibiotic-free blood agar media [Clinical and Laboratory Standards Institute (CLSI), 2015].

### Effects of asclepain cI subinhibitory concentrations (subMICs) on cultures

Effects of asclepain cI subMICs on viability and on the morphology of 13 *H. pylori* strains were determined by viable cell counts and microscopic studies. Aliquots of 14 ml of cultures of each strain were added with 1 ml of the 1 μg/ml subinhibitory concentration (subMIC) of asclepain cI and incubated at 37°C for 24 h. Then, serial dilution (10^−2^ to 10^−6^) viable cell counts were plated in duplicate onto Mueller Hinton Agar (MHA, Britania, Buenos Aires, Argentina) supplemented with 7% horse blood (MHA-HB) and incubated at 37°C for 24 h. Viable cell counts were expressed as colony forming units per ml (CFU/ml). The effect on cell morphology of *H. pylori* in culture treated and untreated with subMICs of asclepain cI was analyzed. Smears were made from the planktonic cultures. Gram stain was subsequently performed. Then, it was observed and photographed under an optical microscope with an immersion objective. In addition, 100 μl of culture was taken, placed on a coverslip, and allowed to dry. Coverslips were gold coated and processed on a standard sputter. Observations were made by scanning electron microscopy (SEM) using a Zeiss LEO 1450VP microscope.

### Gastroprotective effect of asclepain cI

The gastroprotective effect of asclepain cI on the damage induced by intragastrical administration of *H. pylori* was examined. Four groups of teen animals were used. Group 1 was treated with 250 μl of a suspension of *H. pylori* at 1–2 × 10^8^ CFU/mL. Group 2 was treated with 250 μl of asclepain (2 μg/ml), 60 min prior to infection with *H. pylori*. Group 3 was treated with 250 μl of asclepain (2 μg/ml), and Group 4 was treated with 250 μl of phosphate buffered saline (PBS). This procedure was performed for a week every 48 h. The animals were sacrificed by cervical dislocation 4 days after the last inoculation. Lesions in the stomach were observed under an illuminated magnifying microscope, and the number and size of long lesions were both measured. Petechial lesions were counted; five such petechial lesions were taken as 1 mm of ulcer (Awaad et al., 2017).

### Effects of sub-inhibitory concentrations (subMICs) of asclepain cI on the transcription (expression) of *H. pylori* genes encoding pathogenic factors

Gene expression assays were evaluated with SubMICs (½ MIC) of asclepain cI. Total RNA was isolated from cultures of 13 strains with or without enzyme treatment, using TRIZOL reagent according to the manufacturer's instructions (Invitrogen, Buenos Aires, Argentina), being stored at −20°C. The cDNA was obtained as previously described by Salinas Ibáñez et al. (2017).

Reverse transcription of *omp18, ureA, flaA* genes was carried out using 200 U Moloney Murine Leukemia Virus Reverse Transcriptase (M-MLV RT, Invitrogen, Buenos Aires, Argentina). PCR amplification was performed using the primer pairs shown in Table 2 and the protocols described in Table 3.

**Table 2:** Primers used for RT-PCR targeting *Helicobacter pylori* genes.

**Primer**	**Primer sequence (5′-3′)**	**Size amplicon (bp)**
16S *rRNA*-F	GGAGGATGAAGGTTTTAGGATTG	390
16S *rRNA*-R	TCGTTTAGGGCGTGGACT	
*omp*18-F	TGCTTTTGGAAGGCAATACC	165
*omp*18-R	CATTTGGGTTTGGTTTCACC	
*ure*A-F	GCCAATGGTAAATTAGTT	411
*ure*A-R	CTCCTTAATTGTTTTTAC	
*fla*A -F	GTGGCGCAAAAAGTGGCTAA	237
*fla*A-R	GTAATCGGCCGGTTTCAAGC	

**Table 3:** Protocols of PCR amplification for *Helicobacter pylori* genes.

**Steps**	**Temperature (°C)**	**Time (min)**
**16S*****rRNA*** **and** ***omp*****18 genes**		
Initial denaturalization	94	3
30 cycles	94	1
	58	1
	72	1
Final extension	72	10
***ureA*** **and** ***fla*****A genes**		
Initial denaturalization	95	5
	94	1
35 cycles	45	1
	72	1
Final extension	72	7

RT-PCR products were identified in agarose gel electrophoresis (1.8%), stained with GelRed Nucleic Acid Gel Stain (Biotium Inc. Hayward, CA, USA), visualized with UV light, and finally photographed. Molecular mass reference (PBL, Quilmes, Buenos Aires, Argentina) was included. Java image processing and analysis software (ImageJ, Maryland, USA) were used for the semi-quantification of the DNA amplicons.

### Hepatotoxicity and nephrotoxicity assays

The animals used in cytotoxicity tests were those described in point 2.7 [National Institutes of Health (NIH), 1980]. The potential hepatotoxicity and nephrotoxicity of asclepain cl were evaluated using two groups of animals. The first group was treated with an intragastric administration of 1 × PBS. The second group was treated with three doses of 250 μl of asclepain cI (2 μg/ml) at 48 h intervals for 3 days. Then, the animals were sacrificed and the mice serums (before and upon treatment) were collected to determine aspartate aminotransferase (AST), alanine aminotransferase (ALT), and creatinine activity, using Transaminases 200 and Creatinine commercial kits (Wiener Lab, Rosario, Santa Fe, Argentina). The results were expressed as IU/L and mg/L, for aminotransferase and creatinine activity, respectively (American Gastroenterological Association, 2002). Hepatotoxicity was evaluated using AST and ALT assay. The color change produced by the reaction of pyruvate and 2,4-dinitrophenylhydrazine was measured at λ: 505 nm. Nephrotoxicity assays were carried out with serum (100 μl) and 41.4 mmol/L picric acid (500 μl) in a hemolysis tube. The mixture was allowed to stand for 10 min and centrifuged at 3,000 rpm for 5 min. Next, 63 μl of glycine buffer/1.0 M NaOH was added to supernatant (375 μl) and incubated for 20 min at room temperature. The reaction of creatinine with alkaline picrate in a buffered medium produced a chromogenic compound which was measured at λ: 510 nm. A Cintra 2020 UV-Vis Spectrometer (GBS Scientific Equipment Pty Ltd., Braeside, Victoria 3195 Australia) was used for measuring the absorbance.

### Hemolytic activity

The hemolytic activity of asclepain cI was tested against human erythrocytes. Blood was collected with heparin and the erythrocytes were washed and suspended with 35 mM phosphate buffer and 0.15 mM NaCl (pH 7). In total, 500 μl of 0.5% (v/v) of erythrocyte suspension was added to an equal volume of enzyme solutions (2 μg/ml). The mixture was incubated for 1 h at 37°C and centrifuged at 2,800 rpm for 5 min. Hemolysis of the supernatant was determined at λ: 414 nm. Phosphate buffered saline was used as the negative control while Triton X-100 of 0.1% v/v as the positive control (Gonzalez et al., 2010).


(6) $$\begin{array}{c} \text{Hemolysis percentage}=\;\frac{\text{Aa - Ab}}{\text{Ac - Ab}}\;\times \text{100} \end{array}$$

Where:

Aa: Absorbance of asclepain cI.

Ab: Absorbance of the negative control.

Ac: Absorbance of the positive control.

### Statistical analysis

All different determinations, specific enzyme activity, protein content, enzyme physicochemical properties, determination of MIC and MBC, gene expression, viable counts, microscopic techniques, and cytotoxicity test were performed in duplicate as three separate assays. The results were expressed as mean ± standard deviation (SD) using InfoStat/L Statistical Software for Windows (Universidad Nacional de Córdoba, Córdoba, Argentina). A value of *p* < 0.05 was considered significant according to Student's t-test. The linear region of the reaction progress of enzyme activity was also determined.

## Results and discussion

### Protein concentration and specific proteolytic activity

**Figure 1:**
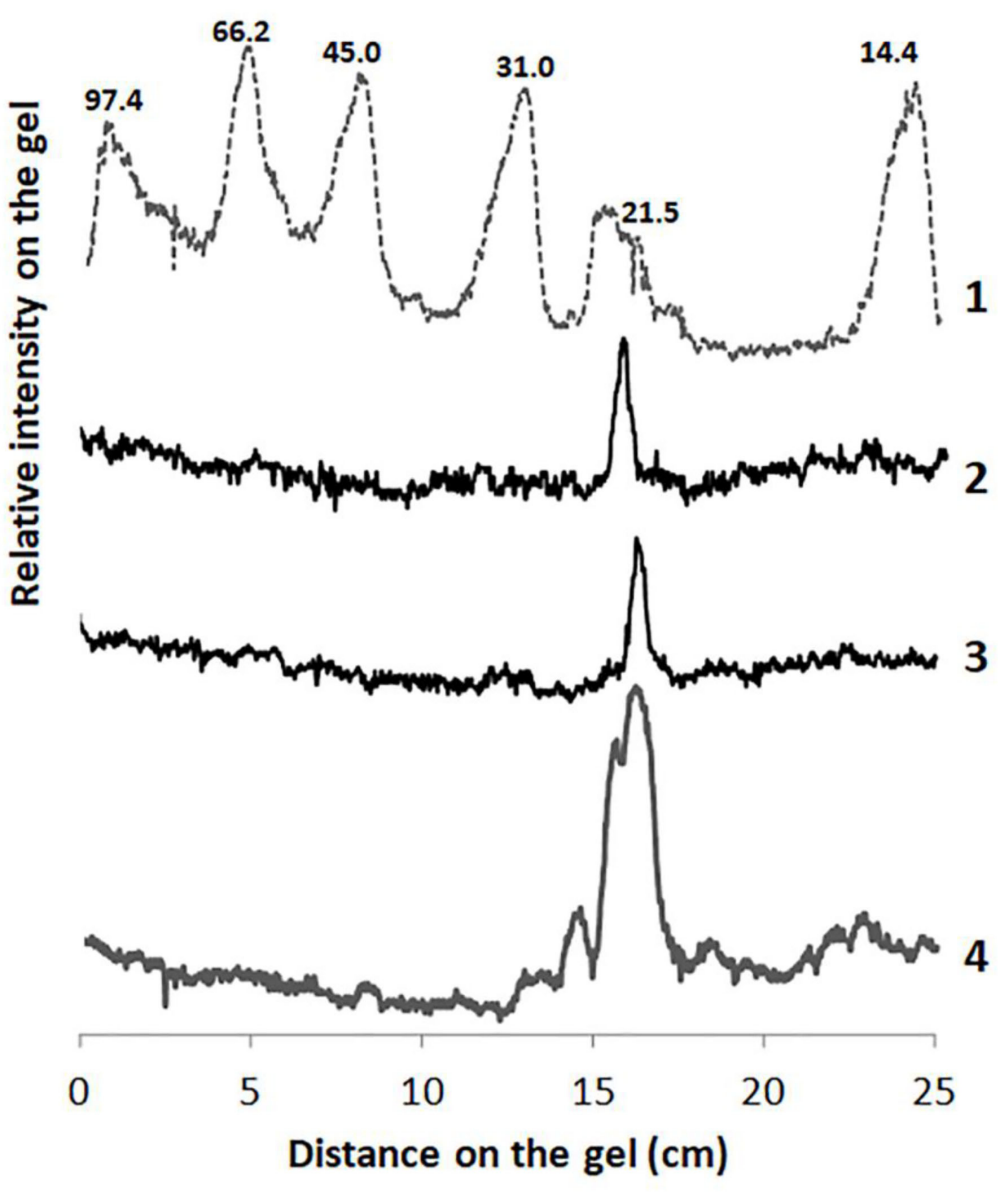
Densitography of SDS-PAGE of *Ascelpias curassavica* proteases. An intensity arbitrary unit (IAU) is plotted as a function of the distance on the gel (cm). Lane 1: Molecular-weight markers (low range kit, BioRad). Lane 2: Asclepain cII. Lane 3: Asclepain cI. Lane 4: Crude extract.

Figure 1 shows the electrophoretic profiles analyzed by densitography using the latest version of ImageJ 1.31. The molecular weights of the two purified fractions from the petioles and stem latex of *A. curassavica*, named asclepain cI and asclepain cII, were 23.422 and 24.653 kDa, respectively. These values were similar to those obtained by MALDI-MS/TOF (Ansari and Yamaoka, 2017; Holmes et al., 2021).

The purified fraction of the asclepain cI showed a total protein content of 135 mg/ml and specific proteolytic activity of 7.74 IU/mg of protein.

### Physicochemical properties of asclepain cI

Asclepain cI was shown to be stable at −20°C for at least 24 months. The enzyme showed a low solubility value (PSI: 0.11 %) and behavior of the Newtonian fluid, with a viscosity of 1.3 cP. Besides, the enzyme showed high water retention (HW: 47 % or WHC: 36.2 g of H_2_O/g of dry residue) and good emulsifying properties (EAI: 3,644 m^2^/g, ESI: 167.6 min). The properties of asclepain cI were similar to those of other foods (Martínez and Carballo, 2021).

### Antibacterial activity of asclepain cI

The antibacterial activity of asclepain cI against *H. pylori* NCTC 11638 (reference strain) and 12 clinical isolates, and the pathologies associated with them, are shown in Table 1 (section Bacterial strains).

All *H. pylori* strains were susceptible to asclepain cI, and MIC values of 1–2 μg/ml and MBC values of 2–4 μg/ml were obtained (Table 4).

**Table 4:** Minimum inhibitory concentration (MIC) and minimal bactericidal concentration (MBC) of asclepain cI against *H. pylori* strains by means of the broth micro dilution method.

***H pylori* strains**	**Pathology**	**Asclepain cI**	
		**MIC**	**MBC**
		**(μg/mL)**	**(μg/mL)**
**Sensitive to AML, MTZ, LEV and CLA**			
NCTC 11638	Reference	2	2
HP155	Chronic gastritis	1	2
HP166	Chronic gastritis	1	2
HP179	Chronic gastritis	1	2
HP659	Chronic gastritis	2	2
**Single-drug resistant**			
HP109	Chronic gastritis R MTZ	1	2
HP137	Chronic gastritis R CLA	1	2
HP145	Duodenal ulcer R MTZ	2	4
HP148	Gastric ulcer R CLA	1	2
HP661	Gastric ulcer R LEV	2	2
HP662	Chronic gastritis R MTZ	2	2
**Multidrug-resistant**			
HP152	Gastric ulcer R MTZ R CLA	2	4
HP294	Duodenal ulcer R MTZ R CLA	2	4

*Inhibition zone diameters to or below 25 mm for AML (10 μg/ml), 28 mm for CLA (15 μg/ml), 18 mm for MTZ (5 μg/ml), and 18 mm for LEV (5 μg/ml) are considered sensitive strains*.

*The values were expressed as mean of the experiments in triplicate (n = 3). No visual color difference was observed between triplicate tests performed under the same conditions*.

The MBC of asclepain cI is 2 μg/ml for all *H. pylori* strains studied, regardless of their sensitivity to antibiotics (CLA, LEV, MTZ, and AML). On the other hand, *H. pylori* HP 145 (resistant to MTZ) and HP 152 (resistant to MTZ and CLA) showed an MBC value as high as 4 μg/ml. Similar behavior was not observed for other *H. pylori* resistant strains, such as HP 109, HP 662 (resistant to MTZ), and HP 294 (resistant to MTZ and CLA).

Several plant extracts used in traditional medicine for gastrointestinal disorders have demonstrated antibacterial activity against *H. pylori* activity. Ethyl acetate extract of leaves and flowers from *Hibiscus rosa-sinensis* L. (Malvaceae) elicited antibacterial and anti-ulcer genic activity, showing MIC of 0.2–0.25 mg/ml and MBC of 1.25–1.5 mg/ml against resistant and sensible *H. pylori* strains (Ngan et al., 2021). Those values were at least 100 times higher than the MIC and MBC values obtained using asclepain cI.

Lien et al. (2019) have reported that ovatodiolide, isolated from *Anisomeles indica*, inhibited the growth of both reference strain and clinical multidrug-resistant isolates of *H. pylori*. The MIC and MBC values ranged from 10 to 20 μM and from 100 to 200 μM, respectively (Lien et al., 2019).

Concentrated ethanol extracts of the entire plant of *A. curassavica* (100 mg/ml) were assayed against 18 bacterial strains by using the agar-diffusion method. Those extracts showed antibacterial activity against *Clostridium histolyticum* ATCC 6282, an anaerobic gram-positive bacterium that can cause gas gangrene in humans and animals, and against *Escherichia coli* ATCC 8739, a gram-negative, facultative anaerobe, no sporulation coliform bacterium. However, the same extracts did neither inhibit the growth of *Bacteroides fragilis* ATCC 23745 (an anaerobic gram-negative bacillus) nor in several gram-positive bacteria, such as *Staphylococcus aureus* ATCC 6538, *S. epidermidis* ATCC 12228, *S. capitis* ATCC 35661, *S. cohnii* ATCC 35662, *Streptococcus pyogenes* ATCC 19615, *S. bovis* ATCC 49133, *S. agalac tiae* ATCC 13813, *S. pneumoniae* ATCC 6303, *S. lactis* ATCC 7962, *Streptococcus sp*. ATCC 12388, *Bacillus subtilis* ATCC 6633, *B. megaterium* ATCC 89, *Corynebacterium diphtheriae* ATCC 13812, and *C. pseudodiphtheriticum* ATCC 10700 (Neto et al., 2002).

In addition, other authors reported that among all the tested species, *E. coli* and *Klebsiella pneumoniae* showed the greatest sensitivity against methanol and petroleum spirit root extracts of *A. curassavica* (Hemadri Reddy et al., 2012). The chloroform extract of *A. curassavica* obtained by the Soxhlet method also showed good activity against the gram-negative bacteria *K. pneumoniae* and *Pseudomonas aeruginosa* but did not show any antifungal activity. The water extract of *A. curassavica* was moderately active against the bacterial strain *P. aeruginosa* and the fungal strain *C. albicans* (Kurdekar et al., 2012). However, those *A. curassavica* extracts have not yet been investigated against *H. pylori*.

In this study, the antibacterial activity of asclepain cI was assayed against *H. pylori* strains that were resistant to one or more drugs. The obtained results demonstrated that asclepain cI exerted significant antibacterial activity against all *H. pylori* strains, including multidrug-resistant strains.

### Effects of subinhibitory concentrations (subMICs) of asclepain cI on cultures

The effect of asclepain cI subMIC (1 and 0.5 μg/ml) on cultures of 13 *H. pylori* strains was evaluated by means of viable cell counts.

A significant decrease in viable cell counts of treated cultures (TP) was regarded as untreated cultures (UTP) (*p* < 0.05). Resistant *H. pylori* strains showed near 2 log units while sensible *H. pylori* strains exhibited 3 log units lesser compared to the control group.

According to literature, 7-O-butylnaringenin (flavonoid) decreased the viable cell count of *H pylori* strains in 2 log units (Moon et al., 2013). Besides, armeniaspirol A, a product isolated from *Streptomyces armeniacus* led to a three-log decrease in viable cell count of *H. pylori* strains (Jia et al., 2022).

On the other hand, we performed both optical and electron microscopy of treated and untreated *H. pylori* cells with asclepain cI.

**Figure 2:**
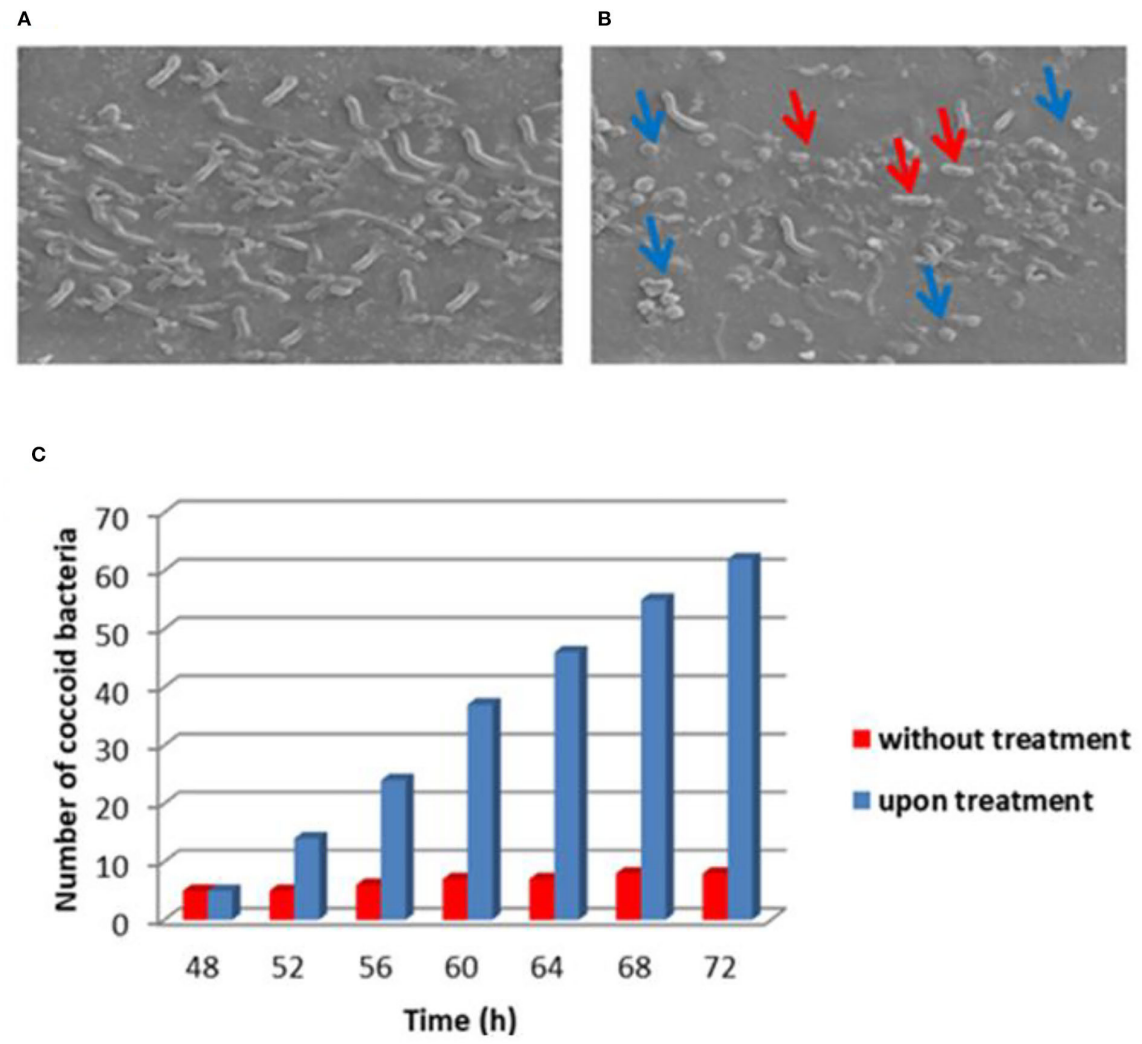
Electron microscopy image of *Helicobacter pylori* NCTC 11638 strain untreated **(A)** and treated **(B)** with asclepain cI. Histogram with statistical analysis of the number of coccoid cells **(C)**.

Figure 2 shows the electron microscopy image of *H. pylori* NCTC 11638 strain untreated and treated with asclepain cI. No evidence of morphological changes or cell damage was observed in the control cultures (without treatment) and the helical shape was maintained. By contrast, the asclepain cI-treated cells showed coccoid (blue arrows) or coccobacilli (red arrows) forms.

The conversion of the helical shape to the coccoid form of the microbial strain hinders the survival and colonization of *H. pylori* in the gastric mucosa.

Other authors have reported that several organic compounds such as methyl gallate, paeonol, 1,2,3,4,6-penta-O-galloyl-β-D-glucopyranose from the root extract of *Paeonia lactiflora*, and the ethyl acetate fraction of the flower from *H. rosa-sinensis* elicited the conversion of helical to coccoid form (Neto et al., 2002; Ngan et al., 2021). The ellagic acid generally found in walnuts, pomegranates, strawberries, blackberries, cloudberries, and raspberries have promoted coccoid morphology in *H pylori* strains (De et al., 2018). In our laboratory, the proteolytic extract of fruits from *Solanum granuloso-Leprosum* and its main purified fraction (granulosain I) both showed similar results (Salinas Ibáñez et al., 2021).

### Effects of subinhibitory concentrations (subMICs) of asclepain cI on the transcription (expression) of *H. pylori* genes encoding pathogenic factors

The effect of subinhibitory concentrations (sub-MICs) of asclepain cI (1 μg/ml) on the expression of the *H. pylori* genes encoding pathogenic factors, such as *omp*18, *ure*A, and *fla*A genes, was determined by using RT-PCR.

**Figure 3:**
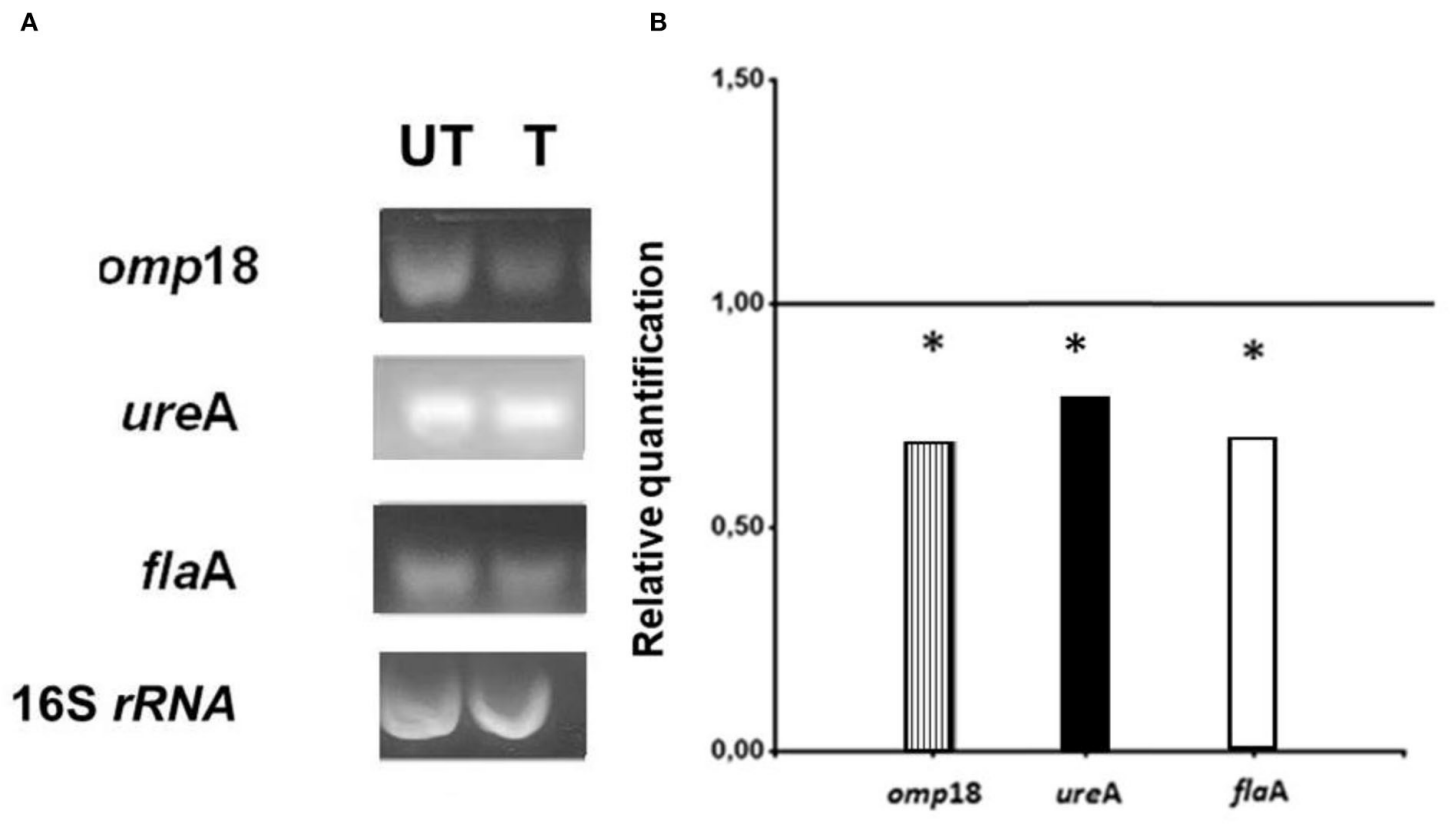
**(A)** Electrophoretic gels: *Helicobacter pylori* amplicons resulting from RT-PCR in 1.8 % agarose gels stained with Gel Red^®^. (T) Treated cultures and (UT) Untreated cultures of *H. pylori* with asclepain cI. **(B)** Relative quantification of the *H. pylori* virulence gene expression, before and after treating *H. pylori* cultures with asclepain cI. The relative quantification represents the mean ± SD of three independent experiments. * *p* ≤ 0.05 according to the Student's *t*-test.

Amplicons of *H. pylori* cultures, which were grown in the presence (T, treated cultures) and in the absence (UT, untreated cultures) of asclepain cI, are shown in Figure 3A.

The expression levels of the *omp*18, *ure*A, and *fla*A pathogenic factors obtained from T and UT were normalized, using the expression level of the 16S rRNA gene (value 1). A comparison was made of the normalized gene expression between treated and untreated cultures and the resulting values were graphed. The mRNA expression levels in *omp*18, *ure*A, and *fla*A significantly decreased in treated cultures (*p* < 0.05) (Figure 3B).

The ability of *H. pylori* to establish a persistent infection depends on the coordinated expression of genes encoding virulence factors that allow the pathogen to adapt to adverse stomach environmental conditions.

The secretion of urease and spiral structure of *H. pylori* are relevant pathogenic factors to establish initial colonization (Matsuo et al., 2017; Ansari and Yamaoka, 2019). Consequently, urease started to be considered an important therapeutic target to explore (Olivera-Severo et al., 2017).

*Helicobacter pylori* has ~4% of the bacterial genome that encodes for a diverse family of outer membrane proteins such as BabA, SabA, OipA, and Omp18. Those proteins facilitate the attachment of bacteria to host cells and help establish persistent infections. In addition, they have an important role in osmotic and structural stability, metabolism, ion transport, and antibiotic resistance (Matsuo et al., 2017; Ansari and Yamaoka, 2019; Šterbenc et al., 2019; Liu et al., 2022).

In *H. pylori*, the *fla*A-encoded flagellin protein is part of the motility organ complex (Kao et al., 2016; Zhao et al., 2019). Evidence-based studies report that the colonization capacity of *H. pylori* is due to the bacillary morphology and the presence of 4–6 unipolar flagella. Additionally, the flagellated strains are correlated with the degree of infectivity and the ability to form bacterial biofilm.

The results obtained suggest that the mechanism of antimicrobial action of asclepain cI is based on the inhibitory effect of the transcription of *H. pylori* genes encoding pathogenic factors.

The literature reports that aqueous extracts of *Lithraea molleoides* (Vell.) Engl. (Anacardiaceae), a regional plant of San Luis Province, Argentina, caused a decrease in the expression of the *ure*A gene of *H. pylori* (Salinas Ibáñez et al., 2017). Besides, the partially purified proteolytic extract of the fruits from *Solanum granuloso-leprosum* and granulosain I (the main purified fraction) significantly decreased the expression of pathogenic factors: *omp*18*, ure*A*, and fla*A (Salinas Ibáñez et al., 2021). Disulfiram, an irreversible inhibitor of aldehyde dehydrogenase (ALDH), also decreased the expression levels of urease from *H. pylori* (Kobatake et al., 2021).

### Gastroprotective effects of asclepain cI

The design of this experiment included four groups of five mice each. The first group consisted of five mice infected with a 1 × 10^6^ suspension of *H. pylori* (Group 1). The second group was of five mice treated with 2 μg/ml of asclepain cI 1 h before being infected with the microorganism (Group 2). The third group included five mice treated with asclepain cI alone (Group 3), and the fourth group consisted of five mice inoculated with PBS (Group 4).

**Figure 4:**
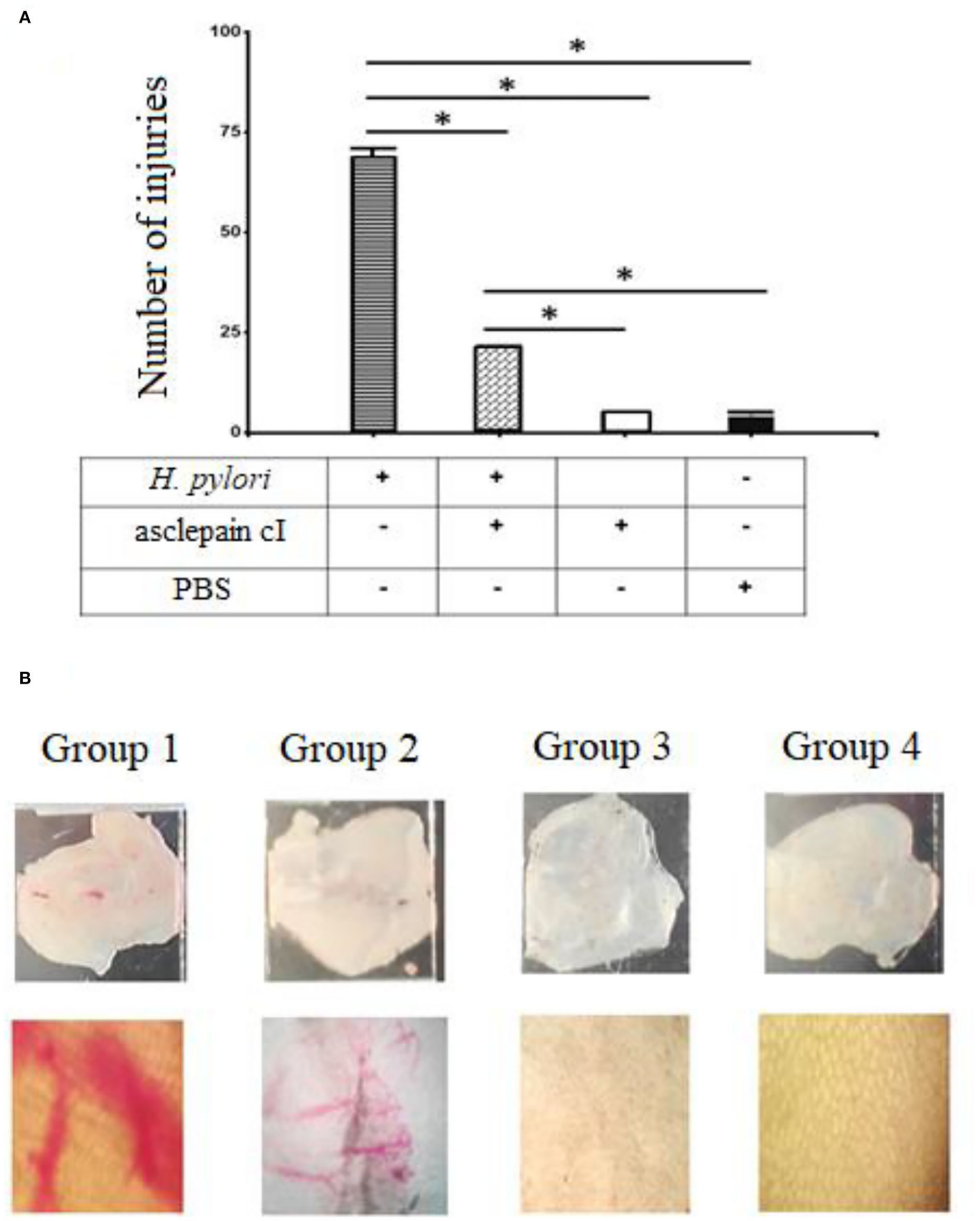
**(A,B)** Gastroprotective effects of asclepain cI. Group 1: Stomach infected with *H. pylori*. Group 2: Stomach treated with asclepain cI and infected with *H. pylori*. Group 3: Stomach treated with asclepain cI. Group 4: Stomach treated with PBS (Control). The * symbol indicates the significant differences between groups (*p < 0.05). All values are expressed as mean ± S.E.M.

Figure 4A shows the number of injuries in the gastric mucosa by direct microscopy (10×) for Group 1 (X: 69), which was significantly higher than the injuries found in the other groups. The differences between groups were significant. Figure 4B shows an image of mucosa after going through different treatments. Asclepain cI showed a noticeable gastroprotective effect.

The mouse infection model has been widely used in the exploration of host responses and eradication of *H. pylori*. A similar gastroprotective effect that ascleplain cI was shown for a tricyclic sesquiterpene extracted from *Pogostemon cablin* (Blanco) Benth (Lian et al., 2018). On the other hand, the gastric mucosal damage caused by *H. pylori* infection was repaired by an extract of *Sanguisorba officinalis* (Shen et al., 2021).

### Cytotoxicity assays

**Figure 5:**
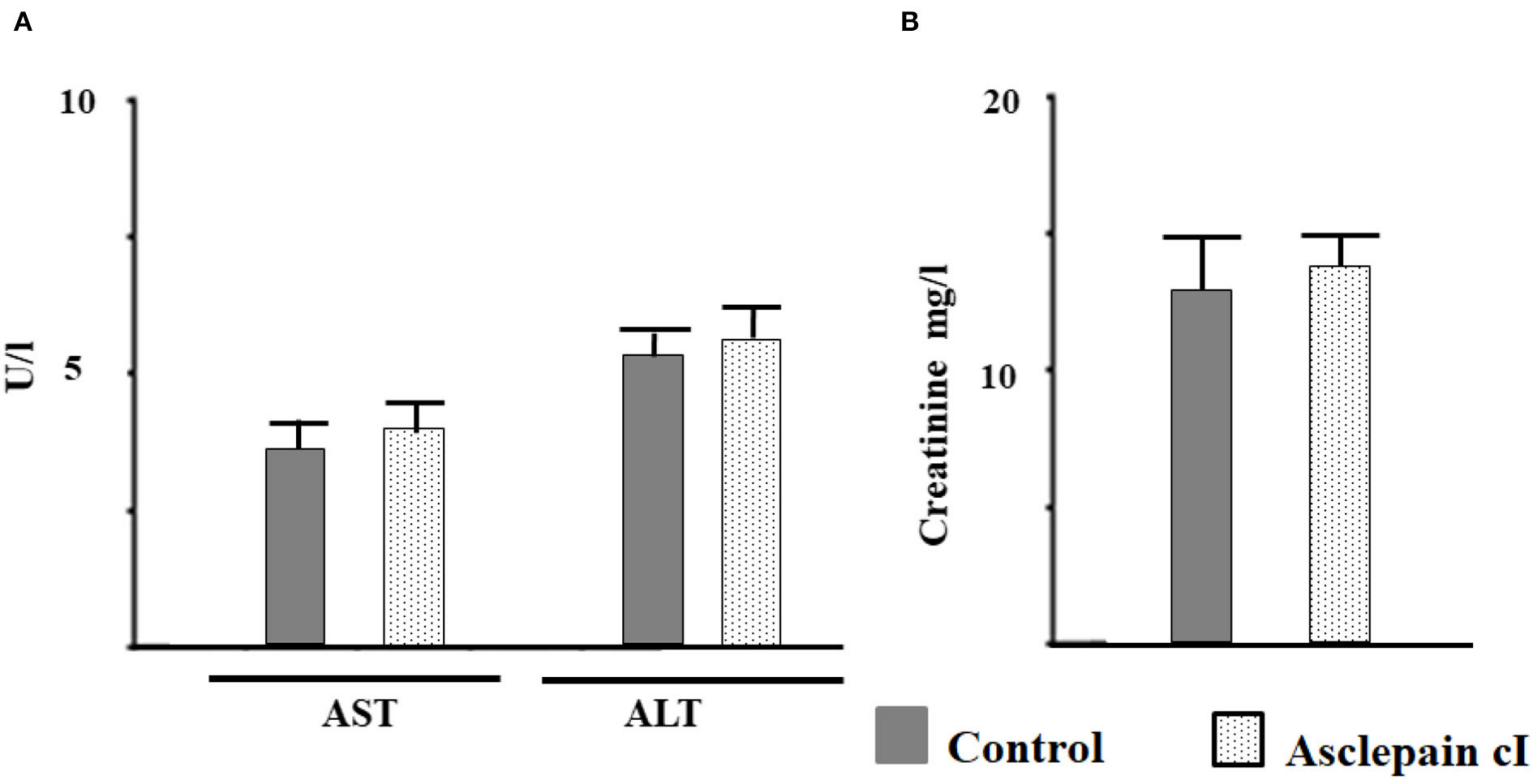
Toxicological effect of asclepain cI evaluated as: **(A)** The activity of aspartate aminotransferase (AST), alanine aminotransferase (ALT); **(B)** creatinine in serum.

The toxicological effect of asclepain cI was evaluated through the determination of the activities of transaminases and creatinine, enzymes involved in liver and kidney function. The activities of these enzymes did not show significant differences (*p* < 0.05) compared to the control group (Figures 5A,B). Consequently, asclepain cI did not show toxicological effects at the concentrations studied. Similar results have been reported with both the partially purified proteolytic extract of the fruits from *Solanum granuloso-leprosum* (Dunal) and the purified fraction named granulosain I, against *H. pylori* (Salinas Ibáñez et al., 2021).

### Hemolytic activity

A hemolytic activity assay is a versatile tool for evaluating the rapid initial toxicity.

The anti-hemolytic activity at the MIC concentration of asclepain cI was 70%. This value shows that asclepain cI has about 1.75–3.5 times better capacity to protect the human erythrocytes than *Chinese keemun black* tea grades (*Camellia sinensis*) (Zhang et al., 2019). It is highly likely that this behavior is based on the ability of the enzyme to form hydrogen bonds with the erythrocyte cell membrane. According to other authors, this interaction can increase the stiffness of the membrane, making it less susceptible to hemolysis (Sato et al., 1993).

## Conclusion

The current persistence and rise of antibiotic resistant bacteria have become a serious concern for global public health, this is all due to the lack of new antimicrobials. Diverse initiatives worldwide yearn to develop novel and more effective antimicrobial compounds and novel strategies. Meanwhile, the potential uses of the compounds found in natural sources for the treatment of *H. pylori* strains are ultimately becoming a safe alternative.

The aim of this paper was to study the effect of asclepain cI, the main purified proteolytic enzyme of the latex of petioles and stems from *Asclepia curassavica* L. (Apocynaceae), a South American native plant, against *H. pylori*, for the purpose of obtaining a natural therapeutic adjuvant and a safe nutraceutical product.

Asclepain cI showed a very good antibacterial activity against 30 sensitive and resistant *H. pylori* strains, with MIC of 1–2 μg/ml and MBC of 2–4 μg/ml.

Besides, asclepain cI significantly decreased the expression of pathogenic factors: *omp*18, *ure*A, and *fla*A. The obtained results allow us to conclude that asclepain cI act on several pathogenic facts which are involved in the structure of the outer membrane protein, in the urea cleavage which allows the bacterium survival, and in the motility of *H. pylori*.

The enzyme showed, on the one hand, a relevant gastroprotective effect in an animal model, and on the other hand, no toxicological effects at the concentrations studied.

Asclepain cI could be successfully used as a natural therapeutic adjuvant against *H. pylori* and also as a safe nutraceutical product.

## Data availability statement

The raw data supporting the conclusions of this article will be made available by the authors, without undue reservation.

## Ethics statement

The animal study was reviewed and approved by Comité Institucional de Cuidado y Uso de Animales, CICUA—UNSL (Institutional Committee for the Care and Use of Animals—National University of San Luis, San Luis, Argentina). Written informed consent was obtained from the owners for the participation of their animals in this study.

## Author contributions

All authors listed have made a substantial, direct, and intellectual contribution to the work and approved it for publication.

## Funding

This work was supported by the National University of San Luis, San Luis, Argentina (Grant Numbers 2-0718 and 02-4118). ÁS and AO are postdoctoral fellows from CONICET, Argentina. SB is a researcher career member at CONICET, Argentina.

## Conflict of interest

The authors declare that the research was conducted in the absence of any commercial or financial relationships that could be construed as a potential conflict of interest.

## Publisher's note

All claims expressed in this article are solely those of the authors and do not necessarily represent those of their affiliated organizations, or those of the publisher, the editors and the reviewers. Any product that may be evaluated in this article, or claim that may be made by its manufacturer, is not guaranteed or endorsed by the publisher.
